# Informed consent for the diagnosis of brain death: a conceptual argument

**DOI:** 10.1186/s13010-016-0042-4

**Published:** 2016-10-13

**Authors:** Osamu Muramoto

**Affiliations:** Center for Ethics in Health Care, Oregon Health and Science University, 3181 S.W. Sam Jackson Park Rd. UHN-86, Portland, 97239-3098 OR USA

**Keywords:** Informed consent, Brain death, Death determination, Jahi McMath, End-of-life decisions, Organ donation

## Abstract

**Background:**

This essay provides an ethical and conceptual argument for the use of informed consent prior to the diagnosis of brain death. It is meant to enable the family to make critical end-of-life decisions, particularly withdrawal of life support system and organ donation, before brain death is diagnosed, as opposed to the current practice of making such decisions after the diagnosis of death. The recent tragic case of a 13-year-old brain-dead patient in California who was maintained on a ventilator for over 2 years illustrates how such a consent would have made a crucial difference.

**Methods:**

Conceptual, philosophical, and ethical analysis.

**Results:**

I first consider a conceptual justification for the use of consent for certain non-beneficial and unwanted medical diagnoses. I suggest that the diagnosis of brain death falls into this category for some patients. Because the diagnostic process of brain death lacks the transparency of traditional death determination, has a unique epistemic structure and a complex risk-benefit profile which differs markedly from case to case, and presents conflicts of interest for physicians and society, I argue that pre-diagnostic counseling and informed consent should be part of the diagnostic process. This approach can be termed as “allow cardiac death”, whose parallel logic with “allow natural death” is discussed. I also discuss potential negative impacts on organ donation and health care cost from this proposal and offer possible mitigation. I show that the pre-diagnostic counseling can improve the possibility for well-thought-out decisions regarding organ donation and terminating life-support system in cases of hopeless prognosis. This approach differs conceptually from the pluralism of the definition of death, such as those in New Jersey and Japan, and it upholds the Uniform Determination of Death Act.

**Conclusions:**

My intention is not to provide an instant panacea for the ongoing impasse of the brain death debate, but to point to a novel conceptual ground for a more pragmatic, and more patient- and family-centered approach. By enabling the family to consent to or decline the diagnostic process of brain death, but not to choose the definition of death, it upholds the current legal definition of death.

## Introduction

Determination of death by neurologic criteria or brain death determination (BDD) has become an integral first step toward harvesting vital organs for transplantation from severely brain-damaged donors. Medical, legal, and bioethical communities worldwide, even in countries such as Israel and Japan where it has traditionally been a cultural taboo, currently accept the use of BDD. Nevertheless, skepticism and confusion about the theory and practice of brain death (BD) have not abated among the general public and even among healthcare professionals [1–4]. A recent high-profile case in California highlighted this confusion about BD and rekindled the debate. Jahi McMath, a then 13-year-old girl, was pronounced brain dead in December 2013 at the Children’s Hospital Oakland following a complication of the surgical treatment of her sleep apnea. Because of the persistent refusal of her family to accept this diagnosis, she has been maintained on “life support”—for more than 2 years as of this writing—in an apartment in New Jersey.

Many comments, criticisms, and words of support about this case have already been published (readers can refer to the case details in [5, 6]). In this essay, I focus on only one question that came up in ethical analysis: What would have happened if the family had been involved and fully informed of the implication of BDD and given a chance to consent *before* proceeding to the diagnostic process? It is unclear from reading various reports whether the family refused the diagnostic process of BDD from the outset, or accepted the diagnostic process, but later rejected the result. Regardless of the scenario, it is almost certain that the family would wish that the patient had never undergone this whole process.

The recent debate on BD in scholarly literature focuses on the issues related to organ donation and the dead donor rule, while BD-related ethical issues outside the organ-donation context are less controversial. BDD is routinely made in intensive care units (ICU) and emergency departments in the process of terminating the life support of hopelessly brain-damaged patients with very little or no approval from the family or surrogate, as seen in the McMath case (hereafter, I will use the term “family” to include other appropriate surrogate decision makers when family members are unavailable). Of course, once BDD is positive, it is a legal death and, technically speaking, the patient (now a corpse) will be quickly placed outside the boundary of health care. While BDD in the context of organ donation is the crucial first step to save other lives, and post-BDD care or “bodily support on a corpse”, is meticulously performed to ensure the freshness of the organs, BDD outside the context of organ donation and rare cases of brain-dead pregnant women is considered just a cut-and-dried legal step to terminate medical care. That is why the McMath case is so unusual in view of this customary practice. While most hospitals have a protocol for removing life support promptly following positive BDD, it is often extremely difficult when the family does not accept such a diagnosis. Some commentators on the McMath case criticized the hospital for its delay in removing life support. For example, one commentator wrote, “… not only is there no need to ask the family’s permission to remove a respirator, but to do so is highly inappropriate” ([5], 380). It was argued that the involvement of the McMath family in Jahi’s case simply served to confuse them further about the status of their child, which, in turn, led to the ensuing dispute. The best course of action, according to this line of thought, is to diagnose BD and discontinue life support unilaterally, regardless of the family’s objections.

While I acknowledge that this is a common practice in many US institutions in recent years, it raises several questions. Even if a grieving period of a few hours is allowed before termination, it precludes any family participation in important end-of-life decisions. Prior to BDD, the medical team engages in patient- and family-centered medical care, and the family’s values and wishes are usually respected, but once BDD is positive, there is no scope for “patient”-centered care because the patient is deceased. While death might be considered as a biological “event,” as seen in the theoretical foundation of BD ([7], 389), death is certainly a long process for many families. They need adequate preparation, and slow and gradual processing after death determination. In a sense, this may be the time when the family wishes to make decisions consistent with their moral and cultural values, more so than when the patient was still alive. Such wishes might include a choice for death by the traditional criteria instead of BD, particularly when the family has not been fully informed of the implications of the BDD and not yet prepared to receive such a diagnosis. However, once BDD has been made it is too late to express any preferences because it is an incontestable medicolegal determination in most jurisdictions, as seen in the McMath case. Therefore, one might ask whether there is any room for including the family in decision making *prior* to BDD. In this essay, I submit an argument that a formal dialogue on crucial end-of-life decisions should take place prior to BDD in the form of a family conference and informed consent.

From the outset, however, I wish to emphasize that this essay proposes consent to the *diagnostic process* of BDD, not to the *definition* of death. In general it may seem odd to consent to a diagnostic process itself. This is because a medical diagnosis is an essential component of patient care and almost always considered beneficial to the patient as long as the diagnosis is used for beneficial care. However, in some special cases, such a diagnosis may not be beneficial or can be harmful to the patient. In such cases, the wishes of the patient and family should be respected before proceeding to a diagnostic process. As discussed shortly, there are already several instances in which potentially harmful diagnoses require informed consent. Is BDD such a case? Another important caveat to be clarified at the outset is that the consent to the criteria of brain death is conceptually and practically different from the consent to the diagnostic process to fulfill the criteria. Obviously, one could accept the criteria, but could still refuse the diagnostic process if there are issues in the process, such as risks and accuracy, that are problematic for the patient and family. This essay addresses the process, not the criteria.

In what follows, I will not plunge into the ongoing fierce debate whether BD is truly human death, but concentrate on ethical dilemmas that necessitate consent prior to the diagnostic process. (For the sake of fairness, I will briefly summarize my position on this debate in the last endnote.) First, I will clarify conceptual issues involved in consenting to medical diagnosis in general, and brain death in particular. Next, I will examine whether consent prior to BDD is feasible, and does not violate the law currently in place, at least in the US. In the following two sections I will address the question of what ethical dilemmas exist that necessitate consent. I demonstrate that the risk-benefit profile of BDD is vastly different from patient to patient, particularly between organ donors and non-organ donors, which requires individualized patient-centered decisions. Other important reasons for honest disclosure and clarification include conflicts of interest, a complex epistemic structure behind the purported perfect accuracy of the diagnosis of BD, and a lack of transparency and perceived diagnostic discrepancy with the reality of the patient’s condition. After the practice of informed consent for BDD is described, two major objections and challenges will be considered next: distributive justice of healthcare resources and potential negative impacts on organ donation. In the final section, I compare this proposal with other pluralistic approaches to legal death, such as those of New Jersey, New York, and Japan. I stress the point that this proposal is *not* to introduce the pluralism of the definition of death, but to present a simple device for consenting to or declining a diagnostic process while upholding the current legal definition of death. As will be mentioned in the end of this article, the author’s intention is not to provide an instant panacea for extremely difficult issues involved in cases like Jahi McMath, but to point to a novel conception for exploring a pragmatic approach to the intractable problems of brain death.

### Conceptual justification for informed consent to medical diagnosis

The first question that needs to be clarified is whether the concept of consent makes any sense for the diagnosis of brain death, and whether it is feasible. I will first consider consent to medical diagnosis in general. Medical diagnosis is a complex process of cognition and action, which consists of several integral steps. First, a clinician has to decide to diagnose or rule out a condition. This step seems banal, but the important point here is that there is always an option for a clinician not to proceed to any diagnostic process.^1^ Second, a clinician performs diagnostic evaluation, such as taking history, examining the patient, and ordering and performing tests. Third, a clinician should decide what diagnostic criteria or algorithm to use to come up with a diagnosis based on the second step. This is closely tied to the second step because in order to establish a diagnosis, an evaluation has to be done to give relevant information required by the criteria. Finally, once a diagnosis is made, it has to be decided whether the patient receives the diagnosis and is treated accordingly, or it is withheld and not used or used for other purposes. Each step is contingent upon the decision of the clinician, which means that there is room, to some extent, for the patient and family to express preferences.

Consent to the first step is usually implied if a patient seeks a diagnosis from a clinician. However, if a diagnosed condition is devastating or fatal with little or no possibility of cure, or if it comes with stigma and discrimination, or if a false positive or negative diagnosis leads to further harm, informed consent and pre-diagnostic counseling are often required before initiating the diagnostic process. Many genetic diagnoses are often of incurable conditions, such as cancer genes and Huntington’s disease, and informed consent is now used routinely. HIV testing used to be in this category, but with improved treatment and prognosis in recent years, the requirement for consent has been gradually relaxed. Of course, in a vast majority of common medical conditions, there is a bona fide agreement that a physician may proceed to a diagnostic process without explicit consent, believing that the benefit from such a diagnosis would outweigh any risks. In terms of the second step (diagnostic evaluation), when the test is invasive and involves risks, it is necessary to seek informed consent except in an emergency. Even if the risk is reasonably small, the patient may still want to decline the diagnostic procedure, such as a colonoscopy, for reasons of pain and discomfort, and informed consent is still indispensable. On the other hand, if the diagnostic evaluation depends on a clinician’s cognitive judgment without involving any risky test, this is usually done without consent. Consent to diagnostic criteria (third step) is indeed an unusual one, and it rarely comes up. In modern medicine, many medical diagnoses are made according to established diagnostic criteria, and most patients and clinicians take it for granted, but if there are different diagnostic criteria available and a patient can be classified differently by different criteria, it is conceivable that the patient may want to consent to or refuse one criterion over another. For example, the diagnosis of multiple sclerosis, or autism spectrum disorder, might be different depending on which criteria are used, particularly for borderline cases. The final step of informing a patient of the diagnosis and using it for other purposes may also require the patient’s involvement. Some patients from a non-Western cultural background request not to receive a serious life-threatening diagnosis such as a cancer diagnosis, particularly when it is terminal, and we generally honor such a request by giving the diagnosis to designated family members. It is also possible that a diagnosis comes up as a result of an evaluation that was done for other purposes. A brain scan done for a research purpose might reveal an incidental brain aneurysm. The disclosure and usage of such a diagnosis obviously requires the patient’s consent.

In the following sections, I will concentrate on the first and second steps (the decision to proceed with, and then execute the diagnostic process) of BDD, and show that consent is feasible and desirable. The third step of choosing the diagnostic criteria of death is, in practice, not something clinicians or families can pick and choose. It is decided by the medical condition leading to death and by the law, and this essay does not intend to propose a legal amendment, as discussed in a later section. There is little or no choice, under the current law and the practice guidelines, for the final step of communicating the result of BDD and applying it as the final diagnosis of death, because positive BDD *is* by definition the diagnosis of BD, which in turn *is* by law the determination of death. There is no room to manipulate or withhold this fact.

### Contingency of brain death and necessity of traditional death

Critics may argue at this point that there is still something odd about consenting to death determination. Generally speaking, with the exception of suicide, death comes to us without our choice or control, and it is usually too late to express any preference for how to die using informed consent when we face imminent death. Physicians do not obtain consent prior to a traditional death determination with cardio-respiratory criteria. Consent makes sense only when a choice for another course of action is available, but death happens almost always as a necessary (inevitable) event without choice, critics might say.

While there is no space for metaphysical discussion of the necessity and contingency of human death, suffice it to say that folk metaphysics appears to accept the metaphysical necessity of death, as seen in the famous adage of “death and taxes”. If the critics can agree that death is a necessary event for every human being (except in religious realms), is BD also a necessary event? In fact, it is quite the opposite. The vast majority of population does not die of BD. BD is a highly contingent event: It is contingent on sustaining severe brain damage with relatively preserved body organs, timely administration of life support and admission to a hospital while the heart is still functioning, a physician’s judgment that the patient likely is brain dead, and most importantly the physician’s chosen action to proceed to the diagnostic protocol of BDD. A traditional death by cardio-respiratory arrest is still necessary for a patient who has contingently died of BD, because the brain-dead body, such as Jahi McMath, is not ready for funeral and burial or cremation. It still has to go through the necessary cardio-respiratory arrest induced by the removal of a ventilator before it can go through normal postmortem confirmation and procedures. This fundamental difference from the traditional death—death that is the final and necessary common pathway to the ultimate non-existence for every human being—makes the informed choice about BD both available and desirable (and at the same time makes an informed refusal of traditional death a logical nonsense). Some might argue that cases of instant death from crushed head or decapitation necessarily die of BD and are counterexamples to my claim. While those cases might have a similar pathophysiology of BD clinically diagnosed in hospitals, they are not cases of BD because BD is, as discussed later, a clinical diagnosis that requires meticulous clinical evaluation following the established protocol. Without this clinical procedure of BDD, the clinical diagnosis of BD is not recognized, even though it is suspected.^2^

Why, then, does the contingency of BD make informed consent available and desirable? Simply put, a contingent event can go otherwise, and if something can go otherwise, there is usually a choice to go otherwise. In fact, many steps leading to BDD are under the control of the medical team and alterable. That is not to say that traditional death by cardio-respiratory criteria allows no choice. There are situations in which cardio-respiratory death (CD or circulatory death) is controlled by the medical team to achieve certain goals and choices are available. The best example is controlled donation after circulatory determination of death (controlled DCDD) [8]. In this protocol, a patient with severe brain damage with extremely poor prognosis (but not brain dead) is removed from a ventilator at the time and place of choice of the medical team (“controlled”), and observed until the heart stops. After a short waiting period following the cardiac arrest, vital organs are harvested for transplantation. This whole process, including the determination of death by circulatory criteria, requires consent of the patient or family because, among other reasons, the timing of CD is contingent upon a decision of the medical team.

The most common choice for CD is between cardiopulmonary resuscitation (CPR, or “full code”) and a do-not-resuscitate (DNR) order. This choice is now routinely given to patients and families in a hospital, particularly when the prognosis is poor. While the main purpose of CPR/DNR is to decide on the use or nonuse of resuscitative efforts and life support systems when the heart and breathing stop, it also gives a chance to decide in which way cardio-respiratory death should be determined. The use of DNR entails a traditional death determined by a *spontaneous* cessation of heartbeat and respiration, which is not intervened by the medical team. In contrast, CPR means that an all-out intervention to reverse cardio-respiratory arrest *must fail* before death is determined. In other words, the determination of death with DNR does not question the *irreversibility* of CD (notwithstanding the legal definition of CD), whereas the determination of death after *attempted and failed* CPR requires the establishment of the irreversibility of cardio-respiratory arrest before CD is pronounced. This choice is given to patients because it is contingent upon the decision and action of clinicians. There is an important parallel between BDD and attempted and failed CPR. BDD determines irreversible cessation of the entire brain function, whereas failed CPR determines irreversible cessation of the cardio-respiratory system. Both establish legal death. Both have alternatives. An alternative to failed CPR is not to do CPR at all, or DNR or “allow natural death” (AND). An alternative to BDD is CD, which should ensue even if BD is not established . This might be called “allow cardiac death”. It is worth reminding ourselves that one of the supporting arguments for equating BD as human death has been that “even with extraordinary medical care, these [somatic] functions cannot be sustained indefinitely—typically, *no longer than several days*” ([President’s Commission [9] p35—emphasis added). Another alternative is an elective termination of life support. However, only CD currently comes with an opt-out option to establish the irreversibility of the lost function. The thesis of this essay is to introduce an option for BDD, comparable with that for failed CPR, to opt out of establishing the irreversible cessation of the function, and to choose the traditional method to determine death.^3^

### Feasibility of informed consent for the diagnosis of brain death

The next consideration for consent to the diagnostic process of BDD is whether there is any room to accommodate such a consent in the middle of extremely critical intensive care. Indeed there is often no choice because if CD comes first, it is impossible to die of BD, and even if BD comes first, if asystole (CD) comes close after BD, there is no time to diagnose BD, let alone consent to the diagnostic process, before CD. Thus, almost every patient’s family accepts their physician’s statement that their relative has deceased by neurologic or cardiac criteria and assumes that there was no choice. However, there are times when options are available between proceeding to the diagnostic process of BDD versus continuing care with the anticipation of approaching CD. Because BD is a highly controlled death in a hospital, there is a unique leeway of several hours to several days between the time of suspected BD and the pronouncement of death. I suggest that this period is an ideal window of opportunity for the family to make important end-of-life decisions. Several factors can influence the decision to proceed or not to proceed to BDD: severity and prognosis of the brain damage, severity and prognosis of systemic organ failure (particularly in the cardiopulmonary system), candidacy for organ donation, the length of stay in the ICU, and family preferences, among others. In current practice, because of the high priority of organ donation and the need to reduce the cost of ICU, BDD is often performed as soon as it is suspected, as exemplified in the McMath case.

It may also be argued that, at least in the US, the choice between consenting or not consenting to BDD is impermissible except for New Jersey and New York, because the Uniform Determination of Death Act (1980) or UDDA [10] allows only one definition that should not be chosen at will. As discussed later in detail, this argument confuses the diagnostic process of BDD and the criteria of BD. This proposal does not let the family choose which criteria of death are to be used. It only lets the family consent to or decline the diagnostic process of BDD for many reasons besides the choice of criteria. However, the family can, of course, still refuse BDD because they refuse to use the criteria of BD. Is this impermissible? The law does not say which criteria must be used under which condition. The major constraint is metaphysical: nobody can die twice by two different criteria under the UDDA. Thus if one is determined dead by neurologic criteria first, that person cannot die by cardio-respiratory criteria later, or vice versa. Whichever comes first, the law only says that the patient is determined dead by *either* brain *or* cardio-respiratory criteria (exclusive disjunction), and that is final. Thus, it is permissible to delay death by CD by artificial means, even if it comes first, and pronounce BD first, which is a routine practice of organ procurement. Likewise, it is permissible to delay death by BD by delaying BDD and pronouncing CD first, which is the current proposal.^4^ In either scenario, whichever death is pronounced first stands as legal death, but there is still room for physicians, in the controlled environment of an ICU, to choose, to a certain extent, which death is to be pronounced first. That is where the input of the family can be incorporated.

### Ethical dilemmas in brain death determination

If informed consent is meaningful and feasible, what are the important ethical dilemmas that necessitate informed consent or refusal of the diagnostic process of BDD? First, let us consider the potential benefit of BDD. If the deceased is a motivated organ donor, death by neurologic criteria fulfills her wishes. For organ donors, a relatively expeditious BDD is needed to accommodate their wishes to donate the freshest organs. If we wait too long without BDD, transplantable organs may deteriorate or the patient may die from CD, and lose a chance to donate organs. If the patient and/or family abhor a prolonged hospital stay in a terminal and unconscious condition, an early diagnosis of BD and the termination of life support would also be consistent with their wishes.

On the other hand, if the patient and family are skeptical about the concept of BD and reject organ donation, like the McMath family, they have nothing to gain by BDD. Their main concern is the prognosis of neurologic damage and survival. They are in absolutely no hurry to receive a diagnosis of death. If anything, they want to continue therapy and delay determination of death as long as possible. Thus, from the perspective of patient- and family-centered care, it is necessary to provide the latest and surest diagnosis of death for non-donors.

Another aspect of the dilemma is the diagnostic accuracy of BD. Organ donors need a high sensitivity or low threshold for the positive diagnosis of BD, while non-donors need a high specificity or high threshold. (This threshold can be adjusted by how strictly the physician adheres to the diagnostic criteria, with the low threshold being less strict adherence, and the high threshold being stricter adherence.) Of course, not all donors want to have an expeditious diagnosis of death, nor do all non-donors want to have a delayed diagnosis of death. The point is that the difference in needs between donors and non-donors for appropriate timing of BDD and the balanced sensitivity and specificity of diagnosis are in opposite directions.

This leads to another crucial ethical dilemma for physicians and hospitals, and perhaps for society in general. They have an obvious vested interest in the early diagnosis of BD because the earlier the BD diagnosis is made, and with a lower threshold for positive diagnosis (or a higher sensitivity), the more they will benefit. Such a diagnosis would result in the increased supply of transplantable organs, a higher turnover rate of expensive ICU beds, and a lower overall cost. Is there any risk of making an early and potentially premature BDD on the part of physicians, hospitals, and society in general? I cannot think of any because such a diagnostic deviation will never be discovered because of the built-in mechanism of the “self-fulfilling prophecy” that leaves no trace behind, as discussed in the next section. Consequently, I submit that it is this conflict of interest, the stark lopsidedness of the risk-benefit balance of BDD—minimal risk and great benefits for physicians, hospitals, and society, versus unknown risk or infinite risk (legal death) and little or no benefit for the patient or the family, particularly those who reject organ donation and BD itself—that justifies, and morally requires, the introduction of informed consent prior to the diagnostic process of BDD.

### Lack of transparency in the diagnosis of brain death

Another important concern for families with a dying loved one is the accuracy and transparency of death determination. The inherent problem of the diagnosis of BD is that the diagnosis is invisible from the layperson’s perspective. Unlike traditional CD in which the family can see, feel, and touch the event of death themselves, BD is hidden from the family’s perception. BDD is usually made retrospectively, but there is no noticeable difference in the patient’s appearance and behavior before and after BDD. This imperceptibility of the diagnosis of BD can engender various emotions in families, including suspicion, skepticism, and the fear of diagnostic error. It is true that CD can also lack transparency when irreversible cardiac arrest is not properly demonstrated. Premature disposal of the dead is a long-standing fear among laypeople throughout human history. That is why every culture has different means to ensure the accuracy of death determination [11]. Many cultures have a wake, or a time immediately following death determination in which family members stay with the deceased, which serves this purpose. By following typical postmortem changes that develop overnight into the next few days, such as algor-, rigor-, and pallor mortis, hypostasis, and putrefaction, the family is reassured that their loved one is ready for burial or cremation. Therefore, families of patients like Jahi McMath, who show no sign of postmortem changes, may be afraid of diagnostic error and the premature termination of life support.

In all fairness, I strongly doubt that there was any significant technical or human error in the diagnosis of the McMath case. Two expert physicians evaluated Jahi at two different time points and reached the same conclusion. Without doubt, she satisfied the criteria. However, this fact is almost irrelevant for skeptical families and the public because the concern over the accuracy of the diagnosis comes from its imperceptible nature and the counterintuitive results of the diagnosis itself, which is never experienced in traditional death.

The difference in the results of death determination between BD and CD is so wide that normal human intuition can hardly comprehend that the current Jahi McMath who seems to be peacefully asleep in an apartment is equivalent to a corpse buried for more than 2 years beneath a tombstone. Media reports from medical and ethics experts invariably emphasized that Jahi’s appearance is merely artificial due to ventilator support and that she is, in fact, a cadaver.^5^ Even so, the public continues to wonder: If the machine can support her for more than 2 years in this condition, how is this condition the same as the traditional death, which no machine can even stop?^6^ Folk metaphysics raises another question that those medical and ethics experts need to respond to: If Jahi’s family had refused the diagnostic process itself and had not received the diagnosis of BD, Jahi today would have been considered alive and would not have been called a cadaver. How could the same and identical Jahi today be both alive and dead depending on whether or not she went through the diagnostic process of BDD?^7^

The diagnosis of BD is a clinical diagnosis without any demonstrable objective data, such as absence of pulse and flat electrocardiogram in the case of CD. Flat electroencephalogram used to be a confirmatory test for BD, but it has become no longer mandatory in the US in recent years [12]. Confirmatory tests in general are discouraged by the leaders of the current practice of BDD [13]. In fact, when there is a discrepancy between a clinical diagnosis of BD and the result of confirmatory tests, there is no other gold standard to decide which one is correct. Thus, confirmatory tests can only aggravate the diagnostic confusion, and those tests have now been renamed ancillary tests. They are used only when clinical evaluation is limited. The concern for diagnostic error also comes from practice variation and protocol violation, which have been discussed in the neurology literature for many years [14, 15]. In one recent study, out of 261 BD cases, 18 % did not adhere to the established guidelines, and neither the apnea test nor another alternative ancillary test were done in 7 % of cases, which is an outright violation of the protocol [16]. The fear of diagnostic error is further fueled by another obscure aspect of BDD, which is the lack of any retrospective confirmation of the accuracy of the diagnosis. Following positive BDD, life support is promptly terminated or vital organs are harvested, which leaves no opportunity for retrospective examination of the accuracy of the diagnosis. Beyond rare exceptions, such as the McMath case, there is no window for retrospective review. This problem has been characterized as a “self-fulfilling prophecy” in the White Paper by the President’s Council on Bioethics ([17], 41–2). In other words, in principle, the current practice of BDD effectively precludes the possibility of discovering diagnostic errors. Furthermore, there are not even any specific postmortem pathological findings [18]. The alleged truth of the diagnosis of BD is necessarily unfalsifiable.

Leaders of the current practice of BDD insist that BDD has “perfect diagnostic accuracy” ([13], 77), but that statement itself is also confusing to families and the public. It does not mean that the diagnosis of BD is correct 100 % of the time against a certain independent gold standard, such as objective laboratory tests, independent retrospective case review, or pathological investigation. It is perfectly accurate because the clinical diagnosis of BD that physicians make based on the established standard is *final* and that is in and of itself the gold standard. In other words, the diagnosis of BD is always correct by default as long as the clinician follows the protocol correctly.

To clarify this type of diagnostic accuracy, I will take an example of the diagnosis of fibromyalgia, which laypeople often see or hear about. Fibromyalgia is a syndrome characterized by widespread body pain, tenderness, fatigue, and many other somatic symptoms, but there is no objective data to confirm this diagnosis, such as laboratory or imaging tests, or pathological examination. The diagnosis is purely clinical and is considered correct when a physician, usually a rheumatologist, determines that the patient fulfils the established diagnostic criteria of fibromyalgia, including physical examination. As in the diagnosis of BD, physical examination requires highly precise evaluation, which includes tender spot examination at 18 specific spots of the body with 4 kg/cm^2^ pressure. The diagnostic criteria are so precise that if the patient has 11 or more tender spots out of 18, the diagnosis is positive, but if she has 10 or fewer, the diagnosis is negative. However, there is no external gold standard to confirm the diagnosis, and it is perfectly accurate by itself as long as the diagnostic protocol is followed correctly. Yet, the disease entity of fibromyalgia continues to be controversial among specialists because, as in the case of BD, there is nothing tangible to indicate the presence of this condition, and empirical research to establish a unifying pathophysiology has been inconclusive [19].

This does not mean, of course, that the diagnoses of fibromyalgia and BD are unimportant or false. In general, clinical diagnoses may not describe the true state of affairs in the patient, but serve as an important explanatory device to describe why the patient is the way he or she is, and as a normative device to advise the patient and the family what to do to relieve or cure the condition ([20], 6–7). A clinical diagnosis of BD explains why the patient’s condition is so hopeless, and provides a normative explanation that this condition is considered equivalent to death, but is optimal for donating vital organs. There is no question about the importance and usefulness of the diagnosis of BD. However, a clinical diagnosis does not necessarily represent accurately what is going on anatomically, biochemically, and physiologically, or the state of affairs in the patient. There is always some discrepancy between a clinical diagnosis and other findings made by other methods. There is no surprise, then, if we see a discrepancy between a BD diagnosis and a finding by another diagnostic test showing, for example, some functioning brain tissue. Likewise, from the layperson’s perspective, the discrepancy between Jahi today in her apartment, which is one end result of the clinical diagnosis of BD, and a buried cadaver, which is the usual end result of the traditional death determination, can be explained by the discrepancy between a contingent clinical diagnosis of BD and the necessary biological and physical reality of traditional death. Alternatively, the fact that her status of death or life depends on whether she underwent the diagnostic process in 2013 demonstrates how BD is, unlike traditional death, contingent on clinical context. I suggest that a lack of such understanding is one of the sources of confusion among the public. The accuracy of the traditional death determination by cardio-respiratory criteria is backed by the visible and palpable reality in the world. This is what the public expect from an accurate death determination, but the accuracy of BDD is considered intrinsic to the diagnosis itself without any extrinsic standard or tangible evidence in the state of affairs in the world. A rare opportunity of retrospective review provided by cases such as Jahi McMath fails to demonstrate the expected postmortem confirmation.

The corollary of the review of the current practice of BDD above is the need for transparency of the diagnostic process of BDD, and providing the family with some sort of tangible evidence for confirmation of death that they can clearly see and accept. One possibility is to invite the family to stay at the bedside to witness the findings of the diagnostic procedure and receive an explanation on the spot. This approach was evaluated by a recent study and showed a significant improvement in the family’s understanding of BD [21]. However, families still require crucial information on BDD *prior* to the commencement of the diagnostic process in order to make important decisions, because once the diagnostic process has started and the result turns out to be positive, the family is already at the point of no return. A positive result entails an immediate and incontestable legal death, and as seen in the McMath case, almost no recourse is left to the family to reverse this determination. In other words, the diagnostic procedure of BD has, besides procedure-related risks that I will describe in the next section, a unique consequence that is of extremely high risk for uninformed families because of its immediate translation into legal death.

### The practice of informed consent prior to the diagnosis of brain death

The important goals of informed consent prior to BDD are the disclosure of relevant and contextualized information on BD, the family’s understanding of the procedure, meaning, risks, and consequences of BDD, and the family’s informed decision to consent or decline. Forms signed by the surrogate may mean nothing unless an honest and complete disclosure, genuine and open-minded conversation and information exchange, and mutual understanding between the physician and the family/surrogate take place. A recent survey of families of pediatric ICU patients who received end-of-life care demonstrated that one of the most important priorities that families request is honest and complete information [22]. Instead of quickly presenting a form and having the family sign it in the waiting room, the informed consent should be performed in a well-prepared family conference with the important stakeholders present. First, the physician must disclose why the procedure is recommended at this time. It is important to ensure a clear understanding of the meaning of BD, which is different from persistent vegetative state or coma. Many surveys show the majority of the public has an erroneous understanding of BD, and this point should be emphasized. It is also important to have the family understand the difference between the irreversible cessation of the function of the whole brain and the structural destruction of the whole brain, which is the key to understanding why imaging studies show a large portion of the brain apparently still intact. Other important points include the following: BDD is a retrospective diagnosis, and once BDD is positive, BD has already occurred even though the family may be unable to perceive any change during that time period; patients with BD usually have a cardiac arrest within several days ([9], 35), although, like Jahi McMath, there are rare cases of individuals who can maintain this condition for weeks to months with a ventilator support [23]; and there is no confirmed case of recovery of consciousness after BDD, though we have limited data because, except for historical cases in 1960’s and 70’s and cases in Japan and New Jersey, almost every case is removed from life support.

It is also crucial that the procedure-related risks be disclosed fully. The main risk comes from the apnea test. Hypotension and cardiac arrhythmia are experienced in 3–33 % of cases [24], which might require the diagnostic procedure to be aborted. Jeret has raised the possibility of informed consent for the apnea test because of the significant risk including death caused by the procedure itself [24]. However, the major risk that leads to immediate death, which the family should fully understand, is the fact that a positive result of BDD means an irreversible and incontestable legal death followed by withdrawal of mechanical ventilation and life support, except when the patient is considered an organ donor or she is pregnant. In most practices, this point seems obvious and tacitly agreed between the physician and the family, but is seldom explained explicitly, much less in writing, before the procedure. While one of the most frequent reasons for proceeding to BDD is to clarify the current status of the patient, it is misleading to say, “we are going to do this test to see if the patient is brain dead or still in a coma and make a care plan accordingly”, because this statement implies that care options are available in either case of the disjunction, when, if BDD is positive, the patient is declared dead and there is no option for further care but to donate organs or to withdraw life support. Thus it is imperative to add, “if this evaluation turns out positive, the only options are to terminate the care or to donate organs because the patient is found legally dead at that point.”

The family should also know that, as discussed above, BDD is a clinical diagnosis and there is no independent gold standard or tangible evidence for the family to confirm the accuracy of this diagnosis. The patient will continue to have heartbeat, respirator-driven breathing, and other signs of life that will not change before and after BDD as long as life support is continued. As with every clinical diagnosis, it is the physician’s judgment that is final, and that result may not necessarily reflect the whole reality of what might happen in the body after death determination. Some might argue that such a statement will undermine the family’s confidence in BDD, but I humbly submit that it is ethically unconscionable to proceed to a death diagnosis while withholding such critical information and keeping the family in the dark. In an expression suitable for the family’s education level and the degree of understanding, the unfalsifiability of the diagnosis of BD should be disclosed. As shown above, this is the fundamental difficulty and the source of confusion in comprehending the equivalence of BD and CD. Otherwise, the public and families continue to be unable to comprehend why Jahi’s current condition is, despite all appearances of life, still considered equivalent to death as they know it.

Contrasting with such caveats for BDD, the physician and the medical team should, in the spirit of shared decision making, also emphasize the positive aspects of BDD and express their recommendations on the values and benefits that the family can draw from BDD. Most importantly, the family can make an informed decision on organ donation, even if it was not considered earlier. While this topic is eschewed before BDD in current practice, I suggest that, if the patient is considered a possible candidate for organ donation, the medical team, if not a transplant coordinator, should disclose in a thoughtful and compassionate manner that BDD is the critical first step for organ procurement. Of course, it is pointless and inappropriate to request organ donation before death is pronounced, but depending on how it is presented and how the family is prepared for such a discussion, this conversation may serve as an important introduction to consideration for organ donation. As discussed earlier, BDD is often needed as soon as it is clinically suspected if the patient is deemed a donor candidate because CD can follow any time and eliminates the chance for donation. This critical fact should not be withheld from the family in the process of consent. Otherwise, the family could erroneously believe that the rash request for the diagnosis of BD is medically necessary for the patient. If the patient is deemed unsuitable as a donor, the family can still come to terms with the decision to withdraw life support if BD is firmly established. The care team may stress this point to undecided family members, though those families should be allowed to take more time if needed than potential donors. Most families are eager to know the prognosis of the loved one and have no difficulty agreeing to BDD after balancing the risks and benefits.

Informed consent is incomplete without presenting available alternatives. A simple alternative is to wait and see, particularly if CD is anticipated within days. This approach may be called “allow cardiac death” as mentioned earlier. It is important to disclose this option because families may not know that it is indeed an option. This option is particularly important for those who are fundamentally opposed to the concept of BD. If the family chose not to agree to BDD at this time, the physician can tell them that the medical team will continue life support for the next day or two and then come back to this question, and ask them to think over this issue, perhaps including the question of organ donation. In the meantime, the physician could stress the fact that the prognosis is extremely poor regardless of BDD, and it is a good time to think about the possibility of an elective withdrawal of life support at the time and place most appropriate for the family and their cultural community. A DNR order and a request for no new life-saving intervention, such as antibiotics, tube feeding, and renal dialysis, along with generous comfort measures, may also become a topic. At this stage, I suggest that the physician refrain from a forced and unilateral diagnostic procedure of BDD against the family’s opposition. The whole purpose of obtaining informed consent is to proceed to the diagnosis only after the family’s understanding. At the same time, the refusal of the family should be interpreted as a context-dependent decision which can be subject to change depending on the next clinical development and further reflection of the family members. Because of the precipitous nature of terminal conditions and the emotional turmoil of the family in those cases, it is hard to predict what will follow next. The patient may die by CD that night. Alternatively, the family may change their mind and agree to a withdrawal of care or proceeding to BDD a few days later, or the patient may improve a little and BD seems less likely next morning. I suggest that the above approach be repeated while incorporating new developments that unfold during the course of the care.

I realize that the above conversation has already taken place informally in many hospitals, but the point I make here is that all such conversations should take place *prior* to BDD, not after in the form of debriefing and negotiation for a withdrawal of life support and organ procurement. In other words, we should invest time and efforts *before* the procedure so that the decisions after the procedure become much easier. Informed consent is meant to be a device to ensure this priority. A family conference held prior to BDD also serves as a pre-diagnostic counseling, and the family’s needs should be addressed compassionately. For those cases without family or an appropriate surrogate decision-maker, a reasonable effort should be made to locate an alternative surrogate decision maker. However, barring any clear indication of cultural and religious convictions (vide infra about New Jersey and New York), the team can proceed to BDD according to an established protocol of each state and hospital for end-of-life decisions for those cases without surrogate decision makers.

For a significant portion of the population who do not believe in the doctrine of BD for various reasons, philosophical, cultural, religious, or otherwise, this informed consent becomes the last chance for patients and families to exercise their dissent. Of course, many among this group may still want to donate their organs, and informed consent can give them a chance to carefully weigh the risks of BD against the benefit of donating organs. However, for those who reject the entire paradigm of BD followed by organ procurement, or BD followed by a withdrawal of life support, and prefer death by the traditional criteria, their wishes and preferences are respected by this informed consent without violating any law. For such families and patients, who are sometimes from religious and cultural minorities, even the small risk of the diagnosis of BD will become infinitely great if the denominator of the risk-benefit ratio, that is, the benefit, is zero. It is important to keep in mind that the risk-benefit ratio is all relative to the patient’s and family’s perspective. The negligible risk of BDD perceived by the medical team and those who accept the current practice of BDD can be felt to be enormous by those who find only risks and no benefit.

### Potential problems of distributive justice

Several side effects can be anticipated from the introduction of informed consent, and I will offer some measures to prevent or mitigate such issues. First, I will examine the problem of distributive justice. In the next section, I will examine its effect on organ procurement. Allowing those hopeless patients without clear diagnosis of death to continue to stay and receive care in the ICU would be very costly, and deprive badly needed ICU beds from those patients who have much more appropriate needs. Furthermore, the critics would argue, even if these patients can be maintained outside ICUs, they will still incur hefty healthcare expenditure, which our society may not be able to support. My response is that, since resource allocation in the current healthcare system is in and of itself an irresolvable ethical issue, I suggest that we need to look at the effect of the proposed informed consent from a wider perspective.

First, it should be noted that, when the Ad Hoc Committee of Harvard Medical School (1968) [25] published a landmark report on BD, there was little guidance to the disposition of those numerous hopeless patients staying in ICUs, and BDD was instrumental in the 80’s through the 90’s in establishing a guidance to terminate care for those most severely brain-damaged cases. However, today the situation is entirely different. It is now almost routine to withdraw life support from many patients with severe devastating conditions with extremely poor prognosis, including permanent unconsciousness, with full family consent regardless of the status of BD [26, 27]. Suspected cases of BD should be treated no differently from other hopeless cases in the ICU in terms of bed allocation. I suggest that we should try an elective withdrawal with full agreement with the family first, which is much more respectful, compassionate, and dignified than unilaterally performing BDD and terminating life support without consent. If CD is expected shortly after BDD, which is often the case, there is no need to proceed to BDD for those who decline consent, because those patients reject organ donation anyway. Of course, this approach by no means excludes an option to proceed to BDD. The point is that the medical team has to have a conversation on this topic with the family first, and find out in which direction the family wishes to proceed. During the course of the dialogue for elective withdrawal, the family might consider BDD as a reasonable justification for withdrawal. In such a case, informed consent is established and BDD should ensue. Alternatively, during the course of such a conversation, the family might decide to donate organs, which also leads to timely BDD with consent.

It is also true that there will always be the exception of families who absolutely refuse BDD or elective withdrawal for cultural, religious, or personal reasons. As usual with those families who insist that physicians “do everything,” it is important to set the goals and limits of medical care, and articulate what they want to accomplish in the hospital within such parameters. I suggest that those cases be consulted with the hospital’s ethics consultation service, and every available options and limitations be considered, including the transfer to a step-down room outside ICU with a DNR order, a transfer to another facility, proceeding to BDD by overriding family’s wishes, or terminating life-saving care through a court order, among others. Some states such as Texas and many hospitals now have explicit policies for such “futile” care, which can be followed.

Finally, statistics from Japan, where BD is used only for organ donors with explicit consent, and the default mode of death is only CD, might be informative [28]. In Japan, most patients with BD remain on life support because they are legally still alive, unless an advance directive and/or the family have consented to organ donation. What happened in Japan is an important showcase of what would happen in the US if we eliminated the above mentioned “self-fulfilling prophecy” paradigm and kept BD cases on ventilators. The Japanese government uses the term “long-term brain death” in its official report, which is defined by “survival” longer than 30 days. Jahi McMath is such a case under these criteria. Two nationwide surveys done in Japan on pediatric cases of BD during the periods of 1987–1999 and 1999–2003 showed that about 21–24 % of BD cases fell into the category of long-term BD, which is much more common in children than in adults [29, 30]. That means that if the US were to keep pediatric brain-dead patients on ventilators, it is possible that roughly one in five cases would have continued to be in this condition for longer than 30 days. However, even though these surveys were not exhaustive, the incidence of such cases per general population in Japan seems extremely low. If we extrapolate their incidence to the US using the ratio of total population between the US and Japan of roughly 2.3 around the years of those surveys, cases like Jahi McMath would occur at most 4 to 9 cases per year in the entire US. Of course, we would never keep all of them on life support, and real numbers are expected to be much lower. Thus, we may be justified in the belief that the use of health-care resources in the US for such patients who opt out of BDD and are treated as a prolonged coma, but in fact are cases of undiagnosed long-term BD, should also be very low. I should also add that Japan spends less than half of the US for healthcare expenditure per capita, even if it keeps all those hopeless cases on life support, and it accomplishes the longest life expectancy and one of the lowest infant mortality rate in the world [31]. As I wrote earlier, the ethics of resource allocation requires a wider perspective than just focusing on a small number of cases with long-term BD.

### Concern about a negative effect on organ procurement

Another important concern is that this proposal might have a negative impact on the willingness of families to donate organs. More generally, the introduction of informed consent prior to BDD might work as a barrier to organ donation and thus decrease the number of donated organs. My response is that this might be the case, but the opposite is also possible. First, informed consent is now ubiquitous in hospitals for so many procedures that most patients and families may not feel anything special about this protocol. Second, if the unwillingness to donate in some parts of the population is based on poor understanding of the reality of BD and organ procurement, as results of a survey of organ procurement coordinators suggest [32], improving the transparency of BDD with full disclosure about the diagnostic process and mutual communication by introducing informed consent and pre-diagnostic counseling should work positively. Third, many organ donors have a greater goal for helping others in need, and informed consent prior to BDD should not present an issue. For those donors, BDD is a necessary part of the whole package of organ donation, and the perceived risk, if any, is offset by the greater benefit of donation. Fourth, for families of prospective organ donors who have not made up their mind, pre-diagnostic counseling and informed consent for BD offer an opportunity to discuss the possibility and benefits of organ donation, as discussed earlier. Such preparatory conversations could achieve a better result for the post-BDD request for donation by an organ procurement coordinator than the current approach. With the current approach, the family is forced to make a difficult decision on donation in a hurry in the wake of the emotional turmoil of the positive BDD without sufficient preparation. With a preparatory discussion prior to BDD, family members can evaluate the issue in detail during the diagnostic process of BDD, and may consequently give a better thought-out decision.

Nevertheless, if a negative impact on organ donation from the full disclosure of BD and the opt-out option of BDD is still a concern, we are faced with another moral dilemma: Is it morally justified to increase the supply of vital organs at the cost of depriving those families of crucial information and their chance for informed decisions, particularly if some of these patients may not need BDD (because CD is imminent, for example), or at least early BDD is unnecessary if they decide not to donate organs, or they want to avoid BD altogether if such a choice is available? In other words, is organ procurement so important that every BD candidate should go through an early diagnostic protocol of BD when it may not be necessary or desired by the patient and family? While an opt-out option for organ procurement has been much debated [33], I contend that an opt-out option for BDD should also be debated in public discourse.

### Comparison with other pluralistic approaches to brain death

The state of New Jersey has an explicit religious exemption from the application of the UDDA [34], and New York requires hospitals to give “reasonable accommodations” for “religious or moral objections” to BD [35]. The most important difference of such accommodations of these states from the current proposal is that they allow two definitions of death used for different populations (religious and others), while the present proposal upholds the current UDDA and only gives a chance to the family to consent to or decline the diagnostic process to fulfill one of the two criteria. As discussed in the second section (conceptual justification), the current proposal involves the options in the first and second steps of the diagnosis of BD (the decision to proceed, and then execute the diagnostic procedure), while the policies in New Jersey and Japan (as I will discuss shortly) involve the options in the third and fourth steps (which criteria to be used and how the result is applied).

Another important difference is, as with any other informed consent, the reason for declining BDD can be anything, whether it is the risk-benefit analysis of the diagnosis itself, the consequence of the diagnosis, or religious, philosophical, or personal reasons. Because informed consent prior to a medical intervention is a well-established medical standard, as opposed to a special exemption only for religious reasons, there is no requirement for a major legal overhaul, whether under state laws or the UDDA.

As described earlier, Japan decided to define BD as human death only in the context of organ donation for the first time in 1997 [28]. The decision on organ donation must come first prior to BDD in a form of an advance directive (donor card, driver’s license, health insurance certificate, or online) which includes the consent to both BD and organ donation, and the family is required to consent in order to proceed to organ procurement.^8^ Death outside the organ-donation program is limited to CD. While this approach is considered by some as allowing a personal choice of the definitions of death [36], it is only within the context of organ donation and must go through rigorous criteria. In other non-donation scenarios, there is no option for patients and families to choose BD as a reason to end futile care with hopeless prognosis. (However, the diagnosis of BD itself for a prognosticating purpose without legal death determination is still permitted, and that is why the incidence of long-term BD cases is known in Japan).^9^

The critical difference of the Japanese system compared with the US system (except for New Jersey) is that Japan has two definitions of death, CD for the general population and BD for consented organ donors, but each group must not use the other group’s definition. This is because, if death were defined as a singular event in Japan as in the UDDA, cases of long-term BD and consented organ donors would have had the same status of life or death. That entails either harvesting vital organs from *living* donors (if CD were the singular definition), or keeping *cadavers* (long-term BD) on ventilators in nursing homes (if BD were the singular definition), which is an utterly unacceptable consequence. Compared with this Japanese approach, the UDDA defines only singular “death” (without qualifying words), but accommodates the pluralism of the *criteria*, not of the definition, in the form of irreversible cessation of *either* “circulatory and respiratory functions” *or* “all functions of the entire brain”. The proposed informed consent only allows families to either consent to or decline a procedure that fulfills one of the two criteria, i.e. neurologic criteria, not the definition, thus upholding the UDDA. If the surrogate of a suspected brain dead patient does not consent to BDD and the patient dies of CD, he died of CD, and did not die of BD first and then CD later. His death is still a singular event under the present proposal. In contrast, an unfortunate situation of Jahi McMath is that she legally died in California, but her status in New Jersey is different. If she eventually undergoes cardiac arrest in New Jersey, she will be pronounced dead in New Jersey by CD, which entails that she dies twice unless the court in California repeals the previous determination of her death.^10^ As Margaret Lock [28] used in her book title, dying twice is one of the problematic consequences of the two-death system, whether in the US or in Japan. I wish to emphasize that the present proposal does not involve any change in the UDDA or a fundamental overhaul of the current foundation of the legal definition of death, but it upholds the present one-death two-criteria system of the UDDA.^11^

The preference of BDD could also be incorporated in an advance directive or a donor card, similar to the Japanese system, but it is unlikely to be used extensively, as the experience in Japan already shows. The most recent nationwide survey done by the Japanese government showed only 12 % of the population registered their donor status (thus consented to or declined BD) through the registration system [37]. The majority of the population, whether in Japan or in the US, are by and large reluctant to complete advance directives, let alone deciding which death criteria they want to choose in a very remote and unlikely scenario of BD. Instead of filling advance directives when they have very little understanding, it is much more relevant and pragmatic to use informed consent and surrogate decision makers when the real need arises and the decision is situated in a real-life contemporaneous context. If anything might be helpful, the public should be encouraged to discuss this topic with family members and prospective surrogate decision makers in advance. Such conversations could eventually enable these proxies to make more relevant and authentic decisions.

## Conclusion

If we strive for patient- and family-centered medical care and shared decision making [27, 38], which I believe we should, I argue that we ought to have a simple and easily accessible device to accommodate the wishes of those who would like to express their unique preferences in the end-of-life decisions at the last moment. This has already been carried out in different forms, including the DNR order and the Physician Orders for Life-Sustaining Treatment (POLST) [39]. I have submitted an argument that a similar measure be taken for the diagnosis of brain death because of its lack of transparency, its unique epistemic structure of the diagnostic reasoning, and the complex risk-benefit profile, as discussed above. I believe that, using informed consent as a guiding topic, a family conference prior to the initiation of the diagnostic protocol of brain death would serve this purpose without requiring a legal overhaul or causing any major disruption in the utilization of healthcare resources and organ procurement. This essay is only a conceptual and ethical argument without any supporting empirical data. This author has no illusion that it provides an instant solution to the decades-long controversy over the theory and practice of BD. Nevertheless, if it opens up a discussion that has not been considered previously, it might become an important step toward improving the understanding and satisfaction of the families of those patients who face imminent brain death.

## Abbreviations

BD: Brain death
BDD: Brain death determination
CD: Cardio-respiratory death
CPR: Cardio-pulmonary resuscitation
DCDD: Controlled donation after circulatory determination of death
DNR: Do-not-resuscitate order
UDDA: Uniform Determination of Death Act

## Footnotes

1 While detailed discussion on the philosophy of medical diagnosis is outside the scope of this essay, I take a position that medical diagnosis is fundamentally a social action, or a social and technological construct ([40], 345). This position contrasts with a prevailing “truth-finding model” of medical diagnosis. Under this traditional model, medical diagnosis is viewed as a physician’s statement of the fact about the “true reality” of the patient. As a “truth-finder”, a physician may feel that diagnosis is her *duty* to uncover whatever pathological reality may exist in the patient. In contrast, under the social-constructivist model, medical diagnosis is performative, rather than constative ([40], 337). It is a *social* and *speech act* that is dependent on the social and techno-historical context of the patient and the diagnostician. With this understanding, medical diagnosis is not much different from any other medical interventions clinicians make on patients, and there is always an option to do differently or not to do at all depending on the clinical context and the patient’s preferences.

2 Here, the metaphysical discussion of medical diagnosis in endnote 1 is relevant again. Undiagnosed and unrecognized state of affairs in human body *may* exist and *can* be uncovered scientifically, but this conjecture should be clearly distinguished from a clinical diagnosis in a specific clinical context, which is totally dependent on diagnosticians. Succinctly put, there is no diagnosis without a diagnostician who diagnoses it within a specific clinical context ([40], 276).

3 This parallel logic between failed CPR and BDD has already been discussed in the President’s Commission Report on defining death in 1981 ([9], 83–4). In this report, this parallel is used to argue that medical intervention should be stopped after BDD, just as we do after failed CPR, because both BDD and failed CPR establish the diagnosis of death. In this report, there is no mention that failed CPR can be opted out whereas BDD can never be opted out. My argument is that as long as failed CPR and BDD equally establish irreversible human death and failed CPR can be opted out through informed consent (which is DNR), BDD could also be opted out through informed consent (which is “allow cardiac death”).

4 It is indeed not uncommon that CD happens while delaying BDD for various reasons, such as trying to correct hypothermia or clear the influence of interfering drug effect.

5 Those who take the “truth-finding model” of medical diagnosis, which I briefly discussed in endnote 1, would insist that Jahi is dead and that is the reality and the “true state of affairs”. According to this received view of medical diagnosis, the “truth of death” is “out there” in the pathophysiology of her brain that is the immutable reality. From the viewpoint of social- and techno-constructivist model of diagnosis, which this author holds, any medical diagnosis is a context-dependent construction of a diagnostician ([40], 321). That means that the diagnosis of BD is a construction of those specific diagnosticians who diagnosed her as such in December 2013. Since this action itself is highly contingent and the diagnosis of BD itself is also highly contingent, as I showed above, the relationship of the present Jahi McMath and her diagnosis in 2013 is also contingent. The corollary of this view is that there is no surprise if Jahi’s current brain does not fulfill the criteria of BD even if it did in 2013.

6 Obviously, our moral intuition differentiates cadavers from human life that is totally dependent on artificial means, such as ventilators and left ventricular assist device (LVAD). If one’s moral intuition tells that a patient in persistent vegetative state is a living human being, most likely it will tell the same for Jahi McMath and others in brain death, as their external behavior is indistinguishable from a persistent vegetative patient who is also paralyzed and ventilator dependent.

7 While space precludes further discussion on this matter, suffice it to say that the very root causes of this confusion are, I believe, the following: first, conflation of the contingent event of BD and the necessary event of human death as a singular event of death; second, ignoring the fact that BD is a distinct *state* with substantial duration of time in which the somatic life is maintained despite the dead brain. The public is well aware that Jahi McMath, brain-dead organ donors being prepared for organ procurement, and brain-dead pregnant women are all maintained in this *state*, despite the experts’ stipulation that BD is a *moment* of death. This is one of the reasons for a commonly used expression in media reports such as, “This patient was diagnosed brain dead two days ago, and today he finally deceased after doctors turned off life support system”.

8 Since 2009, the family may sign the consent even without an advance directive of the patient.

9 If, as in Japan, BDD were not linked with the legal death determination in the US and other western countries, the risk of BDD would have been much more “benign” and BDD could have been done as part of medical investigation without strict consent.

10 According to media reports, the family of Jahi McMath has petitioned the State of California to rescind the death certificate. The court ruling has been pending, but the court granted that it will consider any evidence to prove that she is alive if the family can produce such evidence [41].

11 This statement may seem contradictory to my above-stated criticisms to equating BD as human death. While space precluded a complete discussion of my position on the ongoing debate on whether brain death should be equated to human death, I believe, as thoroughly discussed by other scholars [23, 42, 43], that the current doctrine of equating BD to human death is philosophically and empirically indefensible. In endnote 7, I discussed two critical logical flaws. I also believe, along with other scholars, that the ultimate and logically coherent solution is to treat BD as a clinical *state* that is ideal for organ donation but is clearly distinguished from human death. At the same time, it behooves that the dead donor rule be amended, so that the harvesting of organs from cadavers as well as brain dead *patients* is permitted. The end result would be similar to the current proposal, in that non-organ donors are not automatically removed from life support after positive BDD because they are treated as “not yet dead”, while organ donors can continue to donate freely. The major difference is almost insurmountable challenges of removing the dead donor rule and redefining BD as a separate state from human death, which requires sweeping changes in the public opinion and the law. The present proposal is aimed at achieving a similar result with much fewer changes to the current system.
